# The complement factor 5a receptor 1 has a pathogenic role in chronic inflammation and renal fibrosis in a murine model of chronic pyelonephritis

**DOI:** 10.1016/j.kint.2016.04.023

**Published:** 2016-09

**Authors:** Naheed Choudhry, Ke Li, Ting Zhang, Kun-Yi Wu, Yun Song, Conrad A. Farrar, Na Wang, Cheng-Fei Liu, Qi Peng, Weiju Wu, Steven H. Sacks, Wuding Zhou

**Affiliations:** 1 Medical Research Council Centre for Transplantation, King's College London, Guy's Hospital, London, UK; 2 Core Research Laboratory, the Second Affiliated Hospital, School of Medicine, Xi'an Jiaotong University, Xi'an, Shaanxi, People's Republic of China

**Keywords:** C5aR1, chronic inflammation, pyelonephritis, renal fibrosis

## Abstract

Complement factor 5a (C5a) interaction with its receptor (C5aR1) contributes to the pathogenesis of inflammatory diseases, including acute kidney injury. However, its role in chronic inflammation, particularly in pathogen-associated disorders, is largely unknown. Here we tested whether the development of chronic inflammation and renal fibrosis is dependent on C5aR1 in a murine model of chronic pyelonephritis. C5aR1-deficient (*C5aR1-/-*) mice showed a significant reduction in bacterial load, tubule injury and tubulointerstitial fibrosis in the kidneys following infection, compared with C5aR1-sufficient mice. This was associated with reduced renal leukocyte infiltration specifically for the population of Ly6Chi proinflammatory monocytes/macrophages and reduced intrarenal gene expression of key proinflammatory and profibrogenic factors in *C5aR1*-/- mice following infection. Antagonizing C5aR1 decreased renal bacterial load, tissue inflammation and tubulointerstitial fibrosis. *Ex vivo* and *in vitro* studies showed that under infection conditions, C5a/C5aR1 interaction upregulated the production of proinflammatory and profibrogenic factors by renal tubular epithelial cells and monocytes/macrophages, whereas the phagocytic function of monocytes/macrophages was down-regulated. Thus, C5aR1-dependent bacterial colonization of the tubular epithelium, C5a/C5aR1-mediated upregulation of local inflammatory responses to uropathogenic *E. coli* and impairment of phagocytic function of phagocytes contribute to persistent bacterial colonization of the kidney, chronic renal inflammation and subsequent tubulointerstitial fibrosis.

Urinary tract infections (UTIs) remain among the most common human infectious diseases worldwide (∼150–250 million cases globally per year).^1^, ^2^, ^3^ UTIs present as a wide spectrum of diseases, including bladder infection (cystitis), kidney infection (pyelonephritis), and associated renal damage (e.g., renal fibrosis). Kidney infection is usually caused by ascending infections of the lower urinary tract. Recurrent or long-standing **(**chronic**)** kidney infections can result in renal tubulointerstitial fibrosis, which is a much more common situation in children or in patients with diabetes mellitus or urinary obstructions. Although antibiotics are available to treat the disease, a number of challenges remain, including frequent recurrence, persistence of infection, and the increasing risk for resistance to antibiotics.^1^, ^4^ It is therefore imperative to improve our current understanding of the pathogenesis of UTIs and develop novel therapeutic strategies to improve current treatment.

Uropathogenic *Escherichia coli* (UPEC) is the primary cause of UTIs, and most UPEC express a variety of fimbriae (e.g., P, type 1) that enable them to bind and invade uroepithelial cells.^5^ Although innate immunity plays an essential role in the first line of host defense against pathogens, in UTIs most human UPEC strains are resistant to complement-mediated killing.^6^, ^7^ Bacteria-mediated acute inflammatory responses can cause renal tissue inflammation and epithelium destruction, allowing bacteria to enter the underlying tissue,^8^, ^9^, ^10^ and persistent bacterial colonization and chronic inflammation can lead to tubular atrophy and tubulointerstitial fibrosis.^11^

C5a receptor 1 (C5aR1) is a 350–amino acid glycoprotein and member of the G**-**protein–coupled receptor superfamily of proteins that is expressed in myeloid cells (e.g., neutrophils and monocytes/macrophages [MO/MΦs]) and nonmyeloid cells, including renal tubular epithelial cells.^12^ The well-known ligand for C5aR1 is C5a (also called an anaphylatoxin), which is a 74–amino acid glycopolypeptide fragment generated during complement activation by cleavage of complement C5. The interaction of C5aR1 with C5a mediates a broad spectrum of proinflammatory reactions, such as an increase in vascular permeability, recruitment of leukocytes to sites of injury or infection, generation of cytotoxic oxygen radicals (by granulocytes), and generation of proinflammatory mediators (by myeloid and nonmyeloid cells). A large body of research has demonstrated that C5a/C5aR1 signaling contributes to the pathogenesis of a wide range of inflammatory pathologies, including renal disorders.^12^, ^13^, ^14^ Furthermore, there is compelling evidence from sepsis studies indicating that C5a/C5aR1 signaling can provide counterregulatory effects in host defense through impairment of innate immune cell function and induce excessive inflammatory responses.^15^ Pathogenic roles for C5a/C5aR1 signaling have also been reported in a number of other animal models of infectious disease, such as malaria, acute pneumococcal otitis media, and gram-negative bacteremia.^16^, ^17^, ^18^ However, the roles for C5aR1 in chronic kidney disease, particularly under conditions of infection, are largely unknown.

Given that (i) C5aR1 is expressed in renal resident and inflammatory cells and is up-regulated under pathological conditions,^12^, ^19^, ^20^, ^21^, ^22^ (ii) C5a/C5aR1 signaling is a strong driver of tissue inflammation,^13^, ^14^ and (iii) C5a/C5aR1 signaling has a negative impact on phagocyte function,^23^, ^24^ together with the pathological features of chronic kidney infection (i.e., persistent bacterial colonization, tissue inflammation, and tubulointerstitial fibrosis),^11^, ^25^ we hypothesized that C5aR1 may play a pathogenic role in chronic kidney infection. To test this hypothesis, we used a well-established murine model of chronic pyelonephritis induced by the UPEC strain IH11128 and *C5aR1*-deficient (*C5aR1*^-/-^) mice, as well as a C5aR1 antagonist, to determine the role of C5aR1 in chronic kidney infection (i.e., bacterial load, tissue inflammation, and tubulointerstitial fibrosis). We also investigated the cellular basis of the C5a/C5aR1 axis, which contributes to the pathogenesis of chronic kidney infection, by examining the influence of C5aR1 on cellular infiltration of the kidney following renal infection. In addition, we measured the effects of C5a/C5aR1 on the production of proinflammatory and profibrogenic factors by primary cultured renal tubular epithelial cells (RTECs) and MO/MΦs in response to bacterial stimulation and assessed the impact of C5a/C5aR1 on the phagocytic function of MO/MΦs. Our data demonstrate that following infection, early and persistent bacterial colonization, renal inflammation, and tubulointerstitial fibrosis are dependent on C5aR1, suggesting that C5aR1 facilities the pathogenesis of chronic kidney infection by enhancement of bacterial colonization of tubular epithelium, promotion of local inflammatory responses, and impairment of phagocytic function of MO/MΦs.

## Results

### *C5aR1*^-/-^ mice have reduced bacterial load in the kidney and bladder following bladder inoculation with UPEC

**Figure 1:**
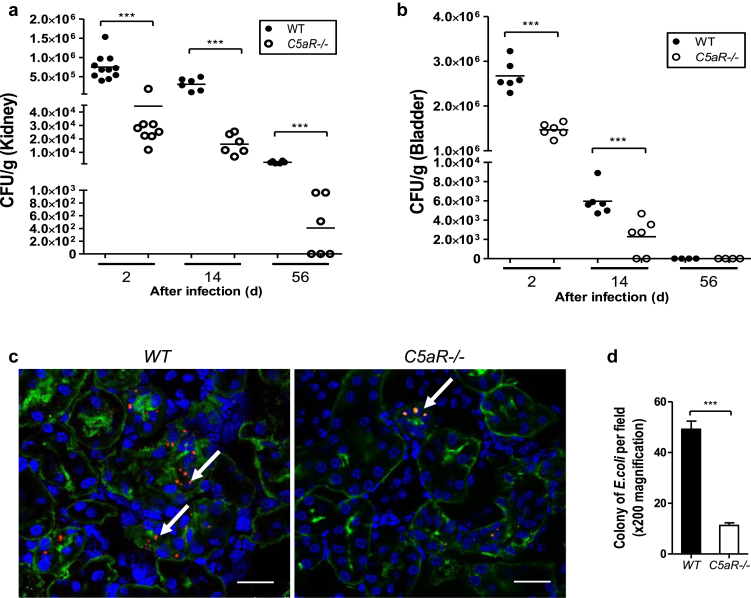
***C5aR1*^-/-^ mice have reduced bacterial load in the kidney and bladder after bladder inoculation with urethropathogenic *Escherichia coli* (UPEC).** Bacterial loads in the kidney (**a**) and bladder (**b**) from wild-type (WT) and *C5aR1*^-/-^ mice were examined at days 2, 14, and 56 after infection. Each dot represents colony-forming units (CFUs) recovered from an individual mouse and is shown as average CFUs from 2 replicate agar plates. Data were analyzed by Student’s *t* test (*n* = 6–11 mice per group). ***P < 0.001. (**c**) Representative fluorescence microscope images of kidney sections from WT and *C5aR1*^-/-^ mice at day after infection, taken at the corticomedullary junction and showing bacterial colonization of renal tubular epithelium (arrows) (blue = 4,6-diamino-2-phenylindole, green = lotus tetragonolobus lectin, red = bacteria) (bar = 25 μm). (**d**) Quantification of bacterial colonies in the kidneys of infected WT and *C5aR1*^-/-^ mice. Data were analyzed by Student’s *t* test (60 viewing fields [×200 magnification] from 4 mice per group). ***P < 0.001. A representative of 2 independent experiments is shown.

Previous studies in a murine model of chronic kidney infection induced by IH11128 have shown that bacterial colonization of the kidney can be detected at 1 to 2 days after infection and persist for months.^11^ We therefore assessed bacterial load in the kidney and bladder of wild-type (WT) and *C5aR1*^-/-^ mice at three stages of infection, namely, early (day 2), intermediate (day 14), and late (day 56) after infection, by counting bacterial colony-forming units (CFUs) recovered from kidney or bladder tissue samples on agar plates.^26^ After bacterial inoculation into the bladder (postinfection), bacterial colonies in the kidney and bladder peaked at day 1 to day 2; afterwords, there was a trend. However, *C5aR1*^-/-^ mice had significantly lower bacterial colony counts in the kidneys at all time points after infection (day 2, day 4, and day 56) and in the bladder at day 2 and day 14 after infection compared with WT mice (Figure 1a and b). We also performed fluorescence microscopy analysis of bacterial colonies in infected kidney tissues from WT and *C5aR1*^*-/-*^ mice at day 2 after inoculation with fluorescence-labeled bacteria. Consistent with the results of the agar plate assay, bacterial colonies in the renal tubular epithelium were significantly lower in *C5aR1*^*-/-*^ mice compared with in WT mice (Figure 1c and d). Collectively, these data demonstrate that C5aR1 deficiency reduces bacterial load in the kidney and bladder.

### C5aR1 deficiency attenuates renal pathology following infection

**Figure 2:**
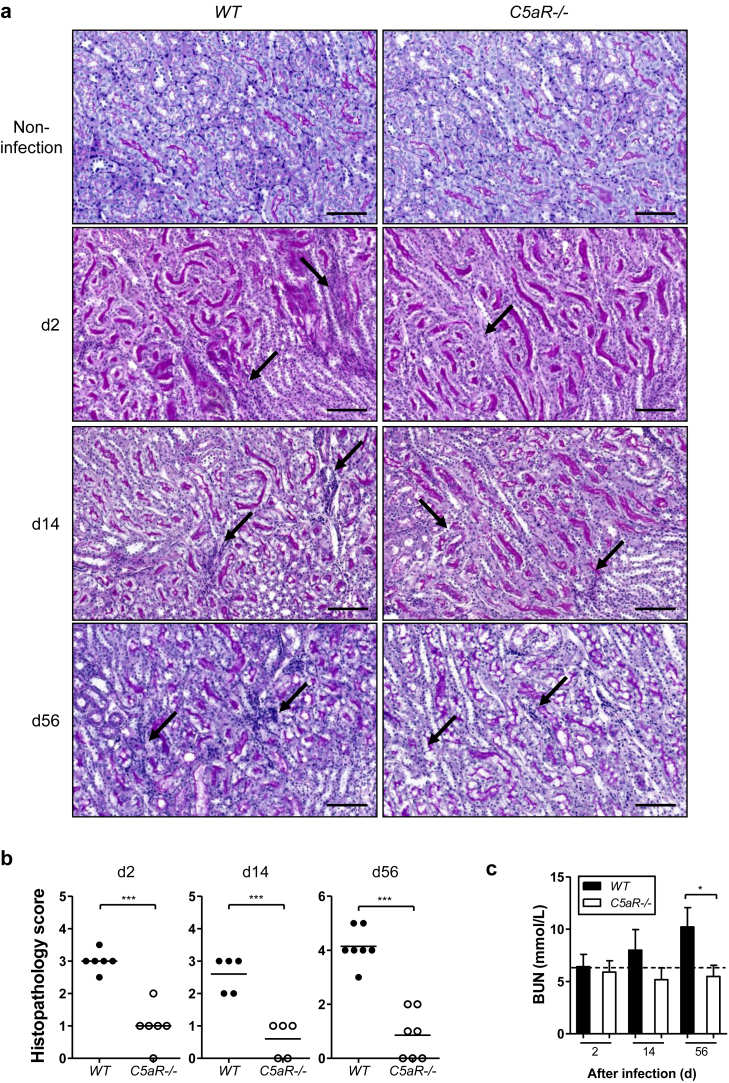
**C5aR1 attenuates renal pathology following renal infection.** (**a**) Representative images of periodic acid-Schiff–stained kidney sections from noninfected and infected wild-type (WT) and *C5aR1*^*-/-*^ mice at days 2, 14, and 56 after infection, taken at the corticomedullary junction. Arrows show renal tubular lesions. Bar = 100 μm. (**b**) Histological scores in the mice illustrated in panel **(a)**. Each dot represents an individual mouse. Data were analyzed by Student’s *t* test (*n* = 5 or 6 mice per group). ***P < 0.001. (**c**) Blood urea nitrogen (BUN) levels in infected WT and *C5aR1*^-/-^ mice at days 2, 14, and 56 after infection. The dotted line on the graph represents BUN level in normal mice. Data were analyzed by Student’s *t* test (*n* = 6–11 mice per group). *P < 0.05. A representative of 2 independent experiments is shown.

The UPEC strain IH11128 used in this study lacks expression of P fimbriae; therefore, they do not cause severe tissue destruction. However, they do mediate the chronic inflammatory process within the renal parenchyma, leading to progressive tissue injury.^11^ We therefore assessed the renal histopathology of infected WT and *C5aR1*^-/-^ mice at different stages of infection. Renal histopathological changes, including cellular infiltration, tubular damage, and interstitial inflammation, were observed at all time points after infection. Tubular atrophy and interstitial inflammation became more apparent at the later time points. The changes were predominantly located within the corticomedullary junction but were also observed in other areas (e.g., the outer cortex and inner medulla). On the basis of these changes, we performed histological scoring of periodic acid–Schiff– and hematoxylin and eosin–stained kidney sections from individual mice in the WT and *C5aR1*^*-/-*^ groups. Compared with WT mice, *C5aR1*^-/-^ mice exhibited attenuated renal histopathological lesions at all time points (days 2, 14, and 56) (Figure 2a and b). We also assessed renal function in WT and *C5aR1*^-/-^ mice after infection by measuring blood urea nitrogen (BUN) levels. Blood urea nitrogen was not significantly elevated in all infected mice when compared with normal mice, but there was a trend toward increased blood urea nitrogen in WT mice at the late time point after infection (Figure 2c). These data demonstrate that renal histopathological lesions and late functional impairment were reduced in *C5aR1*^*-/-*^ mice after infection.

### C5aR1 deficiency influences the extent and phenotype of cellular infiltrates in the kidney in response to infection

**Figure 3:**
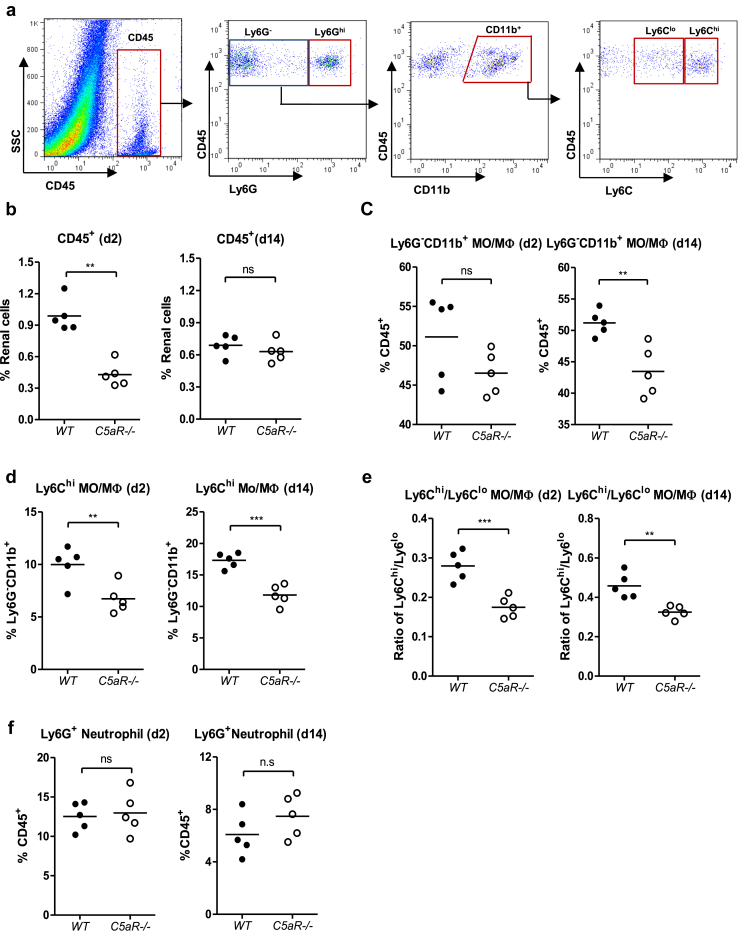

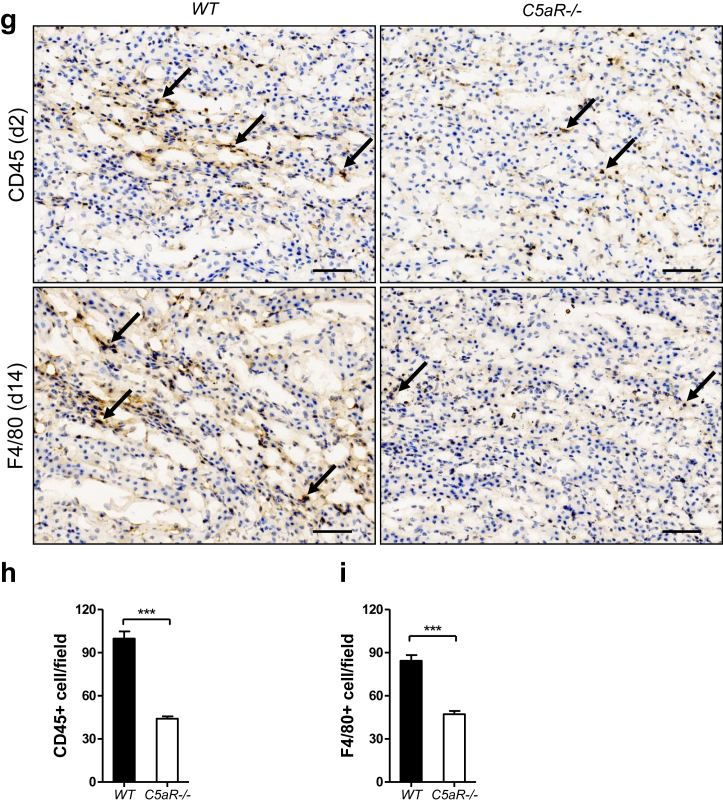
**C5a receptor (C5aR) deficiency influences the extent and phenotype of cellular infiltrates in the kidneys following renal infection.** Renal inflammatory cell infiltration was analyzed in infected wild-type (WT) and *C5aR1*^*-/-*^ mice at days 2 and 14 after infection by flow cytometry. (**a**) Stepwise gating strategy used in flow cytometric analysis of leukocytes, neutrophils, monocytes/macrophages (MO/MΦs), and Ly6c^hi^ MO/MΦs in kidney tissues. (**b–f**) Quantification of leukocytes (CD45^+^), MO/MΦ (Ly6G^-^CD11b^+^), Ly6c^hi^ population, and ratio of Ly6c^hi^ to Ly6c^lo^ populations within the Ly6G^-^CD11b^+^ compartment and neutrophils (Ly6G^+^), respectively. Each dot represents an individual mouse. Data were analyzed by Student’s *t* test (*n* = 5 mice per group). **P < 0.05. ***P < 0.005. (**g–i**) Immunohistochemistry. (**g**) Representative images of CD45- and F4/80-stained kidney sections from infected WT and *C5aR1*^*-/-*^ mice (*n* = 4 mice per group). Arrows show positively stained cells. Bar = 100 μm. (**h,i**) Quantification of CD45^+^ and F4/80^+^ cells. Data were analyzed by Student’s *t* test (40–50 viewing fields [0.04 mm^2^ per field] from 4 mice per group). ***P < 0.001. A representative of 2 independent experiments is shown.

Cellular infiltration was further analyzed by flow cytometry and immunohistochemistry at early and intermediate time points after infection, as described previously.^10^ Flow cytometry analysis of renal cell suspensions showed that when compared with WT, *C5aR1*^*-/-*^ kidneys had lower numbers of leukocytes (CD45^+^) at day 2 after infection and a lower proportion of MO/MΦs (Ly6G^-^CD11b^+^) within CD45^+^ cells at day 14 after infection (Figure 3a–c). In addition, compared with WT, *C5aR1*^*-/-*^ kidneys had a lower Ly6C^hi^ population and reduced ratios of Ly6C^hi^ to Ly6C^lo^ populations within the MO/MΦs (Ly6G^-^CD11b^+^) compartment at both day 2 and day 14 after infection (Figure 3a, d, and e). However, *C5aR1*^*-/-*^ kidneys had a similar proportion of neutrophils (Ly6G^+^) within CD45^+^ cells compared with WT kidneys at day 2 and day 14 after infection (Figure 3a and f). Immunohistochemistry showed that when compared with WT, *C5aR1*^*-/-*^ kidneys had lower numbers of CD45^+^ cells (day 2 after infection) and F4/80^+^ cells (day 14 after infection) than WT kidneys (Figure 3g–3i), which is in agreement with the results of flow cytometry analysis. Collectively, these results indicate that C5aR1 deficiency not only caused a general reduction in cellular infiltration and MO/MΦ accumulation but also specifically reduced inflammatory monocytes' infiltration of the kidney in response to renal infection. However, C5aR1 deficiency did not cause a reduction of neutrophil infiltrate at the time points studied in this model.

### C5aR1 deficiency attenuates renal tissue inflammation and fibrogenesis following renal infection

**Figure 4:**
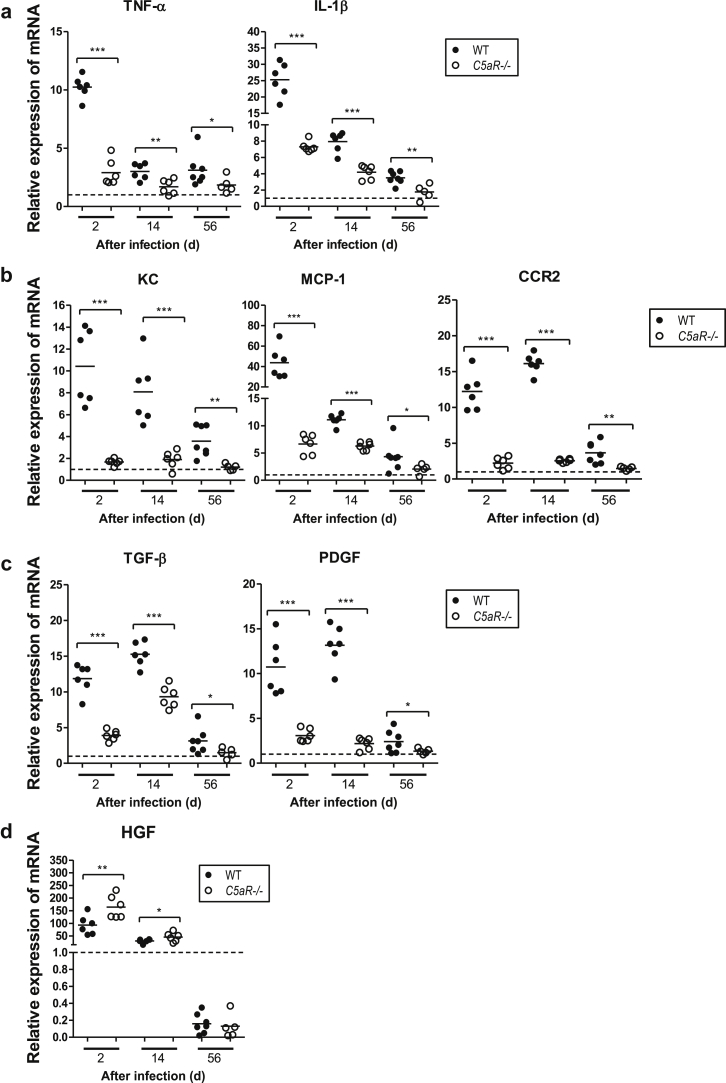
**Absence of C5a receptor (C5aR1) reduces intrarenal gene expression of proinflammatory and profibrogenic factors in response to renal infection.** Relative mRNA levels of proinflammatory and profibrogenic factors in infected kidney tissues from wild-type WT and *C5aR1*^-/-^ mice at the indicated stages of infection by quantitative reverse transcriptase polymerase chain reaction. (**a**) Proinflammatory cytokines (tumor necrosis factor-α [TNF-α] and interleukin-6 [IL-6]). (**b**) Proinflammatory chemokines (keratinocyte-derived protein chemokine [KC] and monocyte chemoattactant protein-1 [MCP-1]) and MCP-1 receptor C-C motif chemokine receptor 2 (CCR2). (**c**) Profibrogenic factors (transforming growth factor-β [TGF-β] and platelet-derived growth factor [PDGF]). (**d**) Hepatocyte growth factor (HGF). Each dot represents an individual mouse and is shown as the mean of 2 replicate polymerase chain reaction results. Data were representative of 3 separate cDNA preparations tested in duplicate for each mouse. The dotted line on the graph represents the gene expression level in normal kidney tissue. Data were analyzed by Student’s *t* test (*n* = 6 mice per group). *P < 0.05, **P < 0.005, ***P < 0.001. A representative of 2 independent experiments is shown.

Persistent tissue inflammation and fibrogenesis play an important role in the progression of chronic kidney disease. We therefore used semiquantitative reverse transcriptase polymerase chain reaction to assess renal tissue inflammation and fibrogenesis of WT and *C5aR1*^-/-^ mice following renal infection. Gene expression of proinflammatory cytokines (tumor necrosis factor-α [TNF-α] and interleukin-1β [IL-1β]), chemokines (keratinocyte-derived protein chemokine [KC], monocyte chemoattactant protein-1 [MCP-1]), chemokine receptor (C-C motif chemokine receptor 2 [CCR2]), and profibrogenic factors (transforming growth factor-β [TFG-β] and platelet-derived growth factor [PDGF]) was significantly reduced in *C5aR1*^-/-^ mice compared with in WT mice at all time points studied (Figure 4a–4c). In contrast, gene expression of antifibrogenic factor (hepatocyte growth factor) was significantly higher in *C5aR1*^-/-^ mice than in WT mice at day 2 and day 14 after infection (Figure 4d). Collectively, these results indicate that absence of C5aR1 attenuates renal tissue inflammation and fibrogenesis following renal infection.

### Less severe renal fibrosis develops in *C5aR1*^-/-^ mice after infection

**Figure 5:**
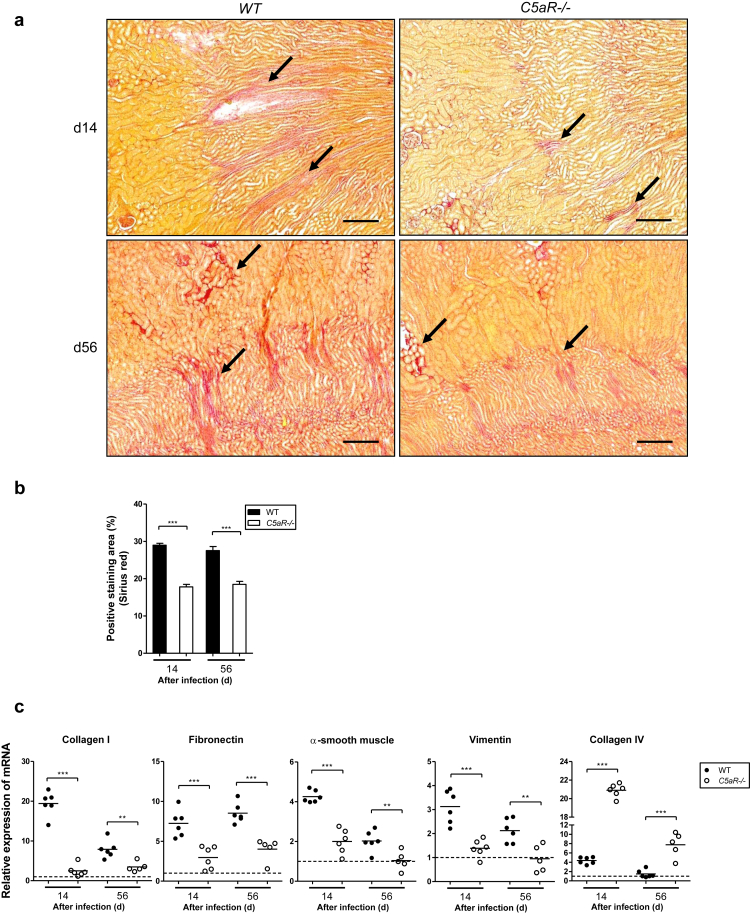
**Less severe renal fibrosis develops in C5a receptor 1–deficient (*C5aR1*^-/-^) mice after infection.** (**a**) Representative images of Sirius red–stained kidney sections from wild-type (WT) and *C5aR1*^-/-^ mice at days 14 and 56 after infection, taken at the corticomedullary junction. Arrows show positively stained area. Bar = 200 μm. (**b**) Quantification of collagen deposition (Sirius red staining) in kidney sections of the mice in panel (**a**). Data were analyzed by Student’s *t* test (20–30 viewing fields [1.92 mm^2^ per field] from 4 mice per group). ***P < 0.001. (**c**) Semiquantitative analysis of mRNA expression of extracellular matrix and cytoskeletal proteins in infected WT and *C5aR1*^-/-^ kidney tissue (*n* = 6 mice per group). Each dot represents an individual mouse and is shown as the mean of 2 replicate polymerase chain reaction results. The dotted line on the graph represents the gene expression level in normal kidney tissue. Data were analyzed by Student’s *t* test. **P < 0.005, ***P < 0.001. A representative of 2 independent experiments is shown.

To further investigate the impact of C5aR1 on the development of renal fibrosis, we assessed the extent of collagen deposition and extracellular matrix production in kidneys from WT and *C5aR1*^-/-^ mice following renal infection. Sirius red staining was performed on kidney sections at day 14 and day 56 after infection. Compared with WT kidneys, *C5aR1*^-/-^ kidneys exhibited a significant reduction of Sirius red staining (Figure 5a and b). We also examined gene expression of several major extracellular matrix and cytoskeletal proteins in kidneys from WT and *C5aR1*^-/-^ mice at day 14 and day 56 after infection using semiquantitative reverse transcriptase polymerase chain reaction. Intrarenal expression of mRNA encoding for extracellular matrix proteins (collagen I and fibronectin) and cytoskeleton proteins (α-smooth muscle and vimentin) was significantly reduced in *C5aR1*^-/-^ mice compared with in WT mice. In contrast, *C5aR1*^-/-^ mice had significantly higher levels of mRNA for collagen IV (which is required for renal tubular epithelial cell structural and functional integrity^27^) than did WT mice (Figure 5c). Taken together, these findings indicate that C5aR1 deficiency protects mice from renal fibrosis and parenchymal loss following renal infection.

### Antagonizing C5aR1 reduces renal inflammation and fibrosis following renal infection

**Figure 6:**
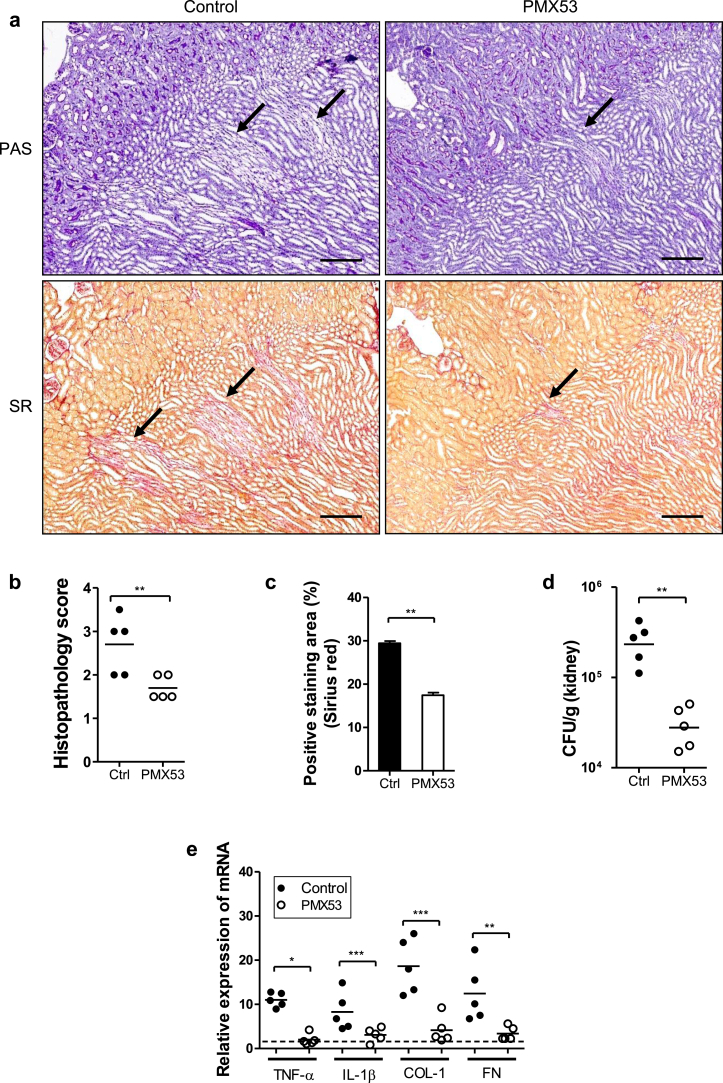

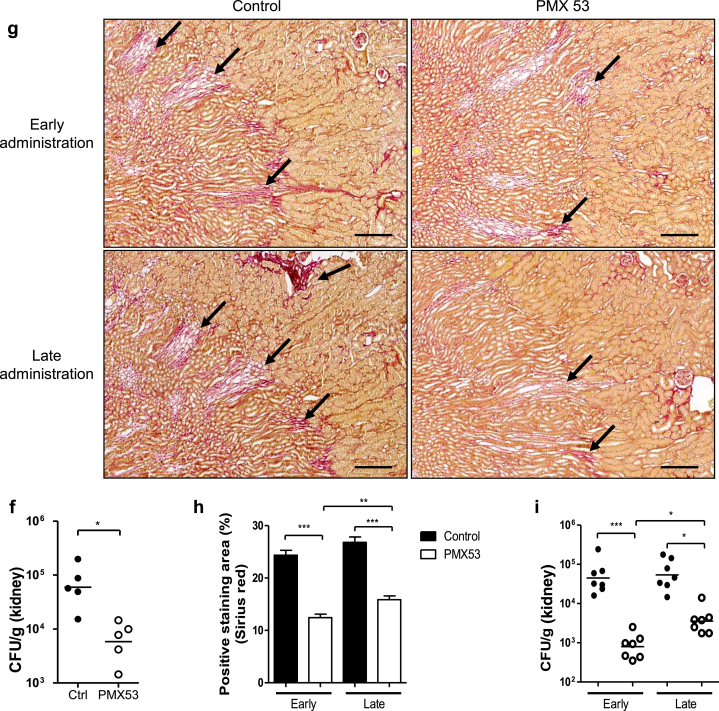
**Antagonizing C5a receptor (C5aR1) reduces renal inflammation and fibrosis following renal infection.** (**a–e**) Wild-type mice were administered PMX53 or control (ctrl) agent daily for 13 days starting on the day of induction of renal infection. Assessment of chronic inflammation, renal fibrosis, and renal bacterial load was performed at day 14 after infection. (**a**) Representative images of periodic acid–Schiff (PAS)- or Sirius red (SR)-stained kidney sections taken at the corticomedullary junction. Arrows show tubular lesions or areas positively stained with Sirius red. Bar = 200 μm. (**b**) Histological scores in the mice illustrated in panel (**a**). Each dot represents an individual mouse. Data were analyzed by Student’s *t* test (*n* = 5 mice per group). (**c**) Quantification of collagen deposition (Sirius red staining) in kidney sections of the mice in panel (**a**). Data were analyzed by Student’s *t* test (20 to 30 viewing fields [1.92 mm^2^ per field] from 5 mice per group). (**d**) Bacterial loads in the kidneys. Each dot represents colony-forming units recovered from an individual mouse and is shown as average colony-forming units from 2 replicate agar plates. (**e**) Semiquantitative analysis of mRNA expression of extracellular matrix and cytoskeletal proteins in infected kidney tissue. Each dot represents an individual mouse and is shown as the mean of two replicate polymerase chain reaction results. (**d**,**e**) Data were analyzed by Student’s *t* test (*n* = 5 mice per group). A representative of 2 independent experiments is shown. (**f–i**) Wild-type mice were given PMX53 or control agent at different stages of infection starting at day 0 (2 hours before the inoculation) and continuing up to day 2 (early administration) or starting at day 3 after the inoculation and continuing up to day 13 (late administration). (**f**) Colony-forming units recovered from the kidney of individual mice at day 3 after infection. Each dot represents an individual mouse. Data were analyzed by Student’s *t* test (*n* = 5 mice per group). (**g**) Representative images of Sirius red–stained kidney sections., taken at the corticomedullary junction. Arrows show area positively stained with Sirius red. Bar = 100 μm. (**h**) Quantification of Sirius red staining in kidney sections of the mice in panel (**g**). Data were analyzed by Student’s *t* test (20–35 viewing fields [1.92 mm^2^ per field] from 5–7 mice per group). (**i**) Colony-forming units recovered from the kidney of individual mice at day 14 after infection. Each dot represents an individual mouse. Data were analyzed by Student’s *t* test (*n* = 7 mice per group). *P < 0.05, **P < 0.005, ***P < 0.001.

In addition to studying *C5aR1*^-/-^ mice, we used a well-known C5aR1 antagonist (PMX53)^28^ to assess whether this approach can curtail renal infection, tissue inflammation, and fibrosis. WT mice were administered PMX53 or control agent daily for 13 days. Consistent with observations made in *C5aR1*^*-/-*^ mice, renal histopathology, tissue inflammation/fibrogenesis, collagen deposition, and bacterial load were significantly reduced in the kidneys of PMX53-treated mice compared with in the control group at day 14 after infection (Figure 6a–e). These results further confirmed the pathogenic role for C5aR1 in this model and also suggest a therapeutic potential for targeting C5aR1 in chronic kidney infection. To further investigate whether the major effects of C5aR1 on chronic inflammation and renal fibrosis are dependent on the initial impact of C5aR1 or the subsequent responses, we performed an additional set of *in vivo* experiments. WT mice were given PMX53 or control agent at the following different stages of infection: (i) starting at day 0 (2 hours before the inoculation) and continuing up to day 2 (early administration) and (ii) starting at day 3 after the inoculation and continuing up to day 13 (late administration). Early bacterial colonization was assessed at day 3 after infection following early administration. Mice that received PMX53 exhibited significantly fewer CFUs in the kidney compared with mice that received control agent (Figure 6f). Chronic injury was assessed at day 14 after infection. Both early and late administration of PMX53 reduced renal fibrosis and tissue bacterial load, but early administration led to a more profound reduction compared with in the control group (Figure 6g–i). These results suggest that the initial impact of C5aR1 plays an important role in the limit of bacterial load and subsequent chronic inflammation and renal fibrosis.

### C5a/C5aR1 interaction amplifies bacteria-induced production of proinflammatory and profibrogenic factors by RTECs and MO/MΦs

Our *in vivo* data presented in this article suggest a critical role for C5aR1 in renal tissue inflammation and fibrogenesis following renal infection. To explore the cellular basis for the C5a/C5aR1 axis contributing to renal tissue inflammation and fibrogenesis, we used primary cell culture systems for RTECs and MO/MΦs and then used reverse transcriptase polymerase chain reaction to assess the effects of C5a/C5aR1 interactions on production of proinflammatory and profibrogenic factors by these cells in response to bacterial stimulation. Incubation with C5a alone led to only a small increase in proinflammatory and profibrogenic factor mRNA expression by RTECs and MO/MΦs. Incubation with heat-killed UPEC alone clearly increased mRNA expression of proinflammatory factors (i.e., tumor necrosis factor-α, interleukin-1β, monocyte chemoattactant protein-1, and keratinocyte-derived protein chemokine) by RTECs and MO/MΦs. In the presence of C5a, mRNA expression levels for those proinflammatory factors were further increased in RTECs and MO/MΦs compared with in the absence of C5a. Combined heat-killed UPEC and C5a treatment also increased mRNA expression of profibrotic factors (i.e., transforming growth factor-β and platelet-derived growth factor) by RTECs and MO/MΦs compared with heat-killed UPEC or C5a treatment alone (Table 1). Thus, our data indicate that engagement of C5aR1 amplifies UPEC-induced production of proinflammatory and profibrogenic factors by RTECs and MO/MΦs.

### C5a/C5aR1 interaction has a negative effect on the phagocytic function of phagocytes

**Figure 7:**
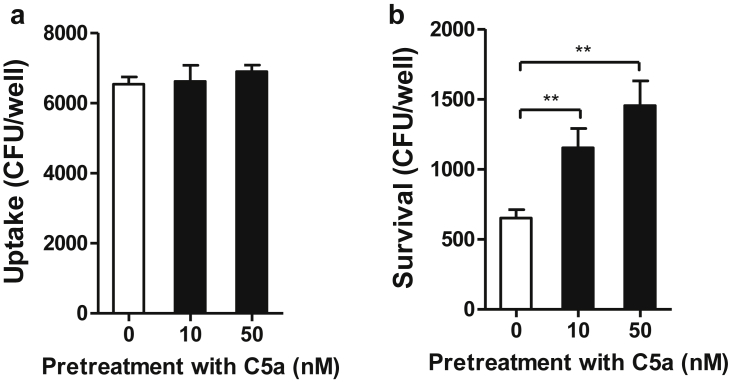
**C5a/C5a receptor 1 (C5aR1) interaction has a negative effect on phagocytic function of phagocytes.** Freshly prepared peritoneal monocytes/macrophages from wild-type mice were pretreated with control agent or C5a (10 nM or 50 nM) for 4 hours and used for uropathogenic *Escherichia coli* phagocytosis and intracellular killing assays. (**a**) Uptake of uropathogenic *E. coli* by monocytes/macrophages. **(b**) Survival of intracellular (phagocytosed) bacteria in monocytes/macrophages. (**a,b**) Data were analyzed by Student’s *t* test (*n* = 5 per group). A representative of 3 independent experiments is shown. **P < 0.005.

In addition to the effects on cytokine production by MO/MΦs, we assessed the impact of C5a/C5aR1 interactions on the ability of phagocytes to kill bacteria by using primary MO/MΦs prepared from WT mice. MO/MΦs were pretreated with C5a (10 nM or 50 nM) for 4 hours and then used for bacterial uptake and intracellular killing assays. The uptake of UPEC was comparable in the control and C5a-treated MO/MΦs, whereas survival of intracellular UPEC was significantly increased in C5a-treated MO/MΦs compared with in the control group, indicating that C5a treatment caused impairment of the bactericidal activity of MO/MΦs but had no apparent effect on bacterial uptake (Figure 7).

## Discussion

Our current understanding of the roles of C5a/C5aR1 in the pathogenesis of kidney disease is primarily based on studies in acute or non–pathogen-related injury. In this study we used a well-characterized murine model of ascending UTI to investigate the role of C5aR1 in chronic kidney injury. Our data demonstrate that C5aR1 deficiency or blockade not only reduces renal bacterial load at all stages of infection but also attenuates tissue inflammation and tubulointerstitial fibrosis, suggesting a pathogenic role for C5aR1 in experimental chronic kidney infection. Mechanistic studies suggest that C5aR1-mediated bacterial colonization of tubular epithelium, persistent local inflammatory responses, and impairment of the phagocytic function of MO/MΦs could contribute to the pathogenesis of chronic kidney infection. Thus, our data further support the notion that excessive or persistent tissue inflammation represents an important pathogenic mechanism in acute and chronic kidney infection.^8^, ^10^, ^11^

One of the important observations in this study is that C5aR1 deficiency or blockade C5aR1 reduced bacterial load at all stages of infection studied, even at the early stage of infection (day 2 or 3 after inoculation). This suggests that C5aR1 could have an impact on early UPEC colonization of renal tract epithelium. In support of this hypothesis, our recent work (in a separate study) has revealed that bacterial adhesion and tissue colonization mediated by the expression of mannosyl residues—a ligand for type 1 fimbriae that we detected on the luminal surface of the renal tubular and bladder epithelium—is increased by C5aR1-induced signaling (unpublished data). In addition to the impact on bacterial colonization, in the present study, we have also found that treatment of MO/MΦs with C5a significantly reduced their bactericidal activity, suggesting that C5a/C5aR1 interaction has a negative regulatory effect on the phagocytic function of MO/MΦs, which is consistent with the well-recognized phenomenon of C5a/C5aR1 signaling being a strong driver of inflammation and having a negative impact on the phagocytic function of phagocytes.^24^, ^29^ Therefore, C5a/C5aR1 interaction–mediated enhancement of UPEC adhesion/colonization of renal tract epithelium (possibly through upregulation of expression of mannosyl residue on the luminal surface of renal tubular and bladder epithelium) and impairment of phagocytic function of phagocytes could contribute to the early and persistent bacterial colonization of the kidney that we observed in this model.

Another important observation from this study is that following renal infection, C5aR1 deficiency or blockade resulted in a reduction in renal tissue inflammation (i.e., cellular infiltration, tubular atrophy, and intrarenal gene expression of proinflammatory factors). These findings strongly suggest that C5aR1 has a critical role in upregulating local inflammatory responses to UPEC, which contributes to chronic inflammation of the kidney and development of renal scarring. To explore the cellular basis of the C5a/C5aR1 axis contributing to tissue inflammation, we examined the impact of C5aR1 on inflammatory cell infiltration and found that *C5aR1*^*-/-*^ deficiency not only reduced the accumulation of leukocytes and MO/MΦs but specifically reduced the population of Ly6C^hi^ proinflammatory MO/MΦs following renal infection. These findings suggest that C5aR1 not only promotes chemotaxis of leukocytes but also plays an important role in modulating the phenotype of infiltrating cells. C5aR1 deficiency resulting in overall inhibition of cellular infiltration and accumulation of proinflammatory MO/MΦs could contribute to the control and resolution of local inflammation.

In addition to cellular infiltration, we also examined the impact of C5a/C5aR1 interaction on cellular responses to UPEC. We focused on RTECs and MO/MΦs, as both types of cells express C5aR1 and also are important sources of proinflammatory and profibrogenic factors in the kidney. Our findings that C5a stimulation up-regulated UPEC-induced production of proinflammatory and profibrogenic factors by cultured RTECs and MO/MΦs suggest that (i) engagement of C5aR1 on both inflammatory and renal parenchymal cells could contribute to local tissue inflammation and (ii) the additive effect of C5a and UPEC on cytokine production by RTECs and MO/MΦs could be explained by the interaction of C5aR1 and Toll-like receptor signaling, as suggested by a previous study in a lipopolysaccharide-induced cytokine production model.^30^

Additionally, our study found that C5aR1 has a big impact on the development of renal fibrosis. Less severe renal tubular interstitial fibrosis developed in *C5aR1*^-/-^ mice than in WT mice following renal infection. The pathogenesis of renal fibrosis is complex, as multiple cell types and molecular pathways are involved. However, it is becoming increasingly clear that the inflammatory microenvironment of the kidney after sustained injury is a key determinant of the dynamic balance between tissue destruction (tubular atrophy and interstitial fibrosis) and repair (tubular cell growth and resolution of renal inflammation and fibrosis).^31^, ^32^ Previous studies have shown that increased intrarenal expression of proinflammatory and profibrogenic factors are strongly associated with renal fibrosis.^32^, ^33^, ^34^ Our finding of an association of reduced renal fibrosis with significant reduction of renal expression of an array of proinflammatory and profibrogenic factors in *C5aR1*^-/-^ mice suggests that C5a/C5aR1-mediated proinflammatory and profibrotic responses could contribute to the pathogenesis of renal fibrosis. In addition, previous studies have shown that the phenotype of the inflammatory cell infiltrate has an impact on chronic renal inflammation and fibrosis, suggesting that renal Ly6C^hi^ proinflammatory MO/MΦs have profibrotic effects. Our findings that *C5aR1*^*-/-*^ deficiency specifically reduced the population of Ly6C^hi^ proinflammatory MO/MΦs following renal infection suggest that C5a/C5aR1 could contribute to the development of renal fibrosis by modulating the phenotype of infiltrating leukocytes.

Intrarenal gene expression profiles from this study revealed that absence of C5aR1 not only reduced the expression of proinflammatory and profibrogenic factors and mesenchymal markers but also led to increased expression of hepatocyte growth factor and collagen IV following renal infection. Hepatocyte growth factor was suggested as an important antifibrotic factor in renal fibrosis, promoting renal cell survival, proliferation, migration, and tubulogenesis and directly antagonizing the profibrotic actions of transforming growth factor-β.^35^ Although the role for collagen IV in renal fibrosis is not well defined, upregulation of intrarenal expression of collagen IV mRNA was found to be associated with reduced renal fibrosis in a unilateral ureteral obstruction model,^36^ thus supporting an antifibrotic role for collagen IV in renal fibrosis. Our finding that C5aR1 has opposing effects on renal expression of profibrogenic and antifibrogenic factors supports the concept that C5aR1 influences the dynamic balance between tissue destruction and repair after renal infection, favoring the promotion of tissue destruction and impairing the repair process.

To conclude, this study is the first to define a novel and important role of C5aR1 in the pathogenesis of experimental chronic pyelonephritis. It also suggests that C5aR1-dependent enhancement of UPEC colonization of renal tract epithelium and excessive local inflammatory responses, as well as impairment of phagocytic function of phagocytes, contribute to the chronic inflammation and renal fibrosis. Our observation that blocking the C5a/C5aR1 axis curtailed the pathology suggests a therapeutic potential for targeting C5aR1 in chronic kidney infection. Although antibiotics can be used to treat the UTI, there is the emerging threat of multidrug-resistant gram-negative bacteria in urology in addition to the well-known frequent recurrence and persistence of infection. Targeting the C5a/C5aR1 axis may offer alternative or combination therapies to reduce the use of antibiotics.

## Methods

### Mice

Homozygous *C5aR1*^-/-^ mice were generated by homologous recombination in embryonic stem cells^37^ and backcrossed onto the C576BL/6 (H-2^b^) parental strain for at least 12 generations. *C5aR1*^-/-^ mice and their WT littermates were used in all experiments, and animal procedures were carried out in accordance with the Animals Scientific Procedures Act 1986.

### Induction of chronic pyelonephritis

A previously described model of ascending UTI leading to chronic pyelonephritis was used. The infection was induced in WT and *C5aR1*^*-/-*^ mice (females 8–10 weeks old) by bladder inoculation of the human UPEC strain IH11128 (10^8^ CFUs in 50 μl phosphate-buffered sailine) *per urethram*. The IH11128 strain (075:K5:H^–^) was isolated from patients with chronic pyelonephritis. It is a mannose-resistant strain expressing both Dr and type 1 fimbriae but lacking P fimbriae and hemolytic activity (a kind gift from Dr. B. Nowicki, University of Texas, Galveston, Texas, USA).^38^ Mice were killed at day 2, day 14, and day 56 after infection for evaluating bacterial load and renal histopathology. In some experiments, tetrarhodamine isothiocyanate–labeled IH11128 was used for imaging bacteria in kidney sections. For C5aR1 antagonist treatment experiments, WT mice were administered C5aR1 peptide antagonist (PMX53, Ac-Phe-cyclo[Orn-Pro-dCha-Trp-Arg]) or control agent (random sequence peptide) (synthesized by GenScript, Shanghai, China) by subcutaneous injection (1 mg/kg daily) starting at different stages of infection and for different times.

### Assessment of renal histopathological features

Paraffin sections (4 μm) were stained with hematoxylin and eosin, periodic acid–Schiff stain, or Sirius red. Stained kidney sections were scanned with a Hamamatsu Nanozoomer 2.0 HT slide scanner (Hamamatsu Photonics, Hamamtsu, Japan) and viewed using NDP.view2 software. Renal histopathological changes were assessed on periodic acid–Schiff– and hematoxylin and eosin–stained sections using a 6-point scale in which 0, 1, 2, 3, 4, and 5 indicated normal, very little, very mild, mild, moderate, and severe histological lesions, respectively. The assessment was based on histopathological changes (i.e., cellular infiltration, tubular atrophy, and interstitial inflammation) that were mainly located at the corticomedullary junction area. Renal fibrosis was assessed on Sirius red–stained sections. The positively stained areas were quantified by imaging analysis (ImageJ software; National Institutes of Health, Bethesda, MD, USA). Briefly, 6 to 8 corticomedullary junction viewing fields selected from appropriate areas within each kidney were examined. Positively stained areas were expressed as a percentage of the whole field area (1.92 mm^2^). All the aforementioned quantitative analyses were performed in a blinded fashion by 2 experienced persons.

### Measurement of bacterial load in the kidney and bladder

Total bacterial load in kidney and bladder tissue was measured by the agar plate assay as previously described, with modifications.^26^ In brief, the tissue was weighed and subsequently homogenized in 2 ml (for the kidney) or 1 ml (for the bladder) of phosphate-buffered saline. A quantity of 100 μl of a series dilution of homogenates were plated on cysteine-, lactose-, and electrolyte-deficient plates. After incubation of the plates for 24 hours, bacterial CFUs on the agar plates were manually counted and expressed as CFU per gram of tissue.

### Statistical analysis

Data are shown either as the mean ± SEM or the readout of individual mice. Unpaired Student’s *t* test was used for comparison between two groups. All the analyses were performed using Graphpad Prism Software version 5 (GraphPad Software, LaJolla, CA, USA).

## Disclosure

All the authors declared no competing interests.

## Figures and Tables

**Table 1:** C5a/C5aR1 interaction amplifies bacteria-induced production of proinflammatory and profibrogenic factors by RTEC and MO/MΦ

mRNA	RTECs^a^			MO/MΦs^a^		
C5a	UPEC	C5a/UPEC	C5a	UPEC	C5a/UPEC
TNF-α	1.1 ± 0.1	5.1 ± 1.2^c^	17.4 ± 3.3^c^^,^^e^	1.1 ± 0.3	4.0 ± 1.0^b^	6.7 ± 1.0^b^^,^^e^
IL-6	2.0 ± 0.1^b^	48.3 ± 9.9^c^	77.3 ± 8.7^d^^,^^e^	2.9 ± 1.9	14.8 ± 3.1^c^	34.2 ± 5.4^c^^,^^e^
MCP	1.9 ± 0.3^b^	48.2 ± 8.3^d^	78.1 ± 10.1^c^^,^^e^	2.1 ± 0.4^b^	2.7 ± 0.5^b^	4.6 ± 0.6^d^^,^^e^
KC	1.2 ± 0.1	14.1 ± 2.8^c^	121.1 ± 16.1^d^^,^^e^	1.8 ± 0.2^b^	1.9 ± 0.1^b^	6.9 ± 1.5^b^^,^^e^
TGF-β	1.2 ± 0.2	0.8 ± 0.3	2.8 ± 0.7^b^^,^^e^	2.2 ± 0.4^b^	1.8 ± 0.2^b^	5.7 ± 0.9^c^^,^^e^
PDGF	1.4 ± 0.2	1.0 ± 0.2	3.5 ± 0.6^c^^,^^e^	1.4 ± 0.2	1.4 ± 0.2	4.2 ± 0.7^c^^,^^e^

Ca5R1, C5a receptor 1; IL-6, interleukin-6; KC, keratinocyte-derived protein chemokine; MCP, monocyte chemoattactant protein-1; MO/MΦ, monocyte/macrophage; PDGF, platelet-derived growth factor; RTEC, renal tubular epithelial cell; TGF-β, transforming growth factor-β; TNF-α, tumor necrosis factor-α; UPEC, uropathogenic *Escherichia coli*.

a Primary cultured RTECs and freshly prepared peritoneal MO/MΦs from wild-type mice were incubated with heat-killed UPEC and C5a, alone and combined, for 24 hours and subjected to reverse transcriptase polymerase chain reaction analysis. mRNA expression was expressed as fold change over the control (absence of C5a and UPEC). Superscript letters b–d indicate significant difference versus control (^b^*P* < 0.05 ^c^*P* < 0.005 ^d^*P* < 0.001).

e Significant difference versus UPEC alone (*P* < 0.05). Data were analyzed by Student’s *t* test (*n* = 5 per group). A representative of 3 independent experiments is shown.

## Supplementary Material

Supplementary Methods The following additional information for methods is given in supplementary methods: materials, cell cultures, assessment of effects of C5a/C5aR1 interaction on cytokine production by RTECs and MO/MΦs in response to bacteria stimulation, assessment of bacterial uptake and intracellular killing by MO/MΦs, assessment of renal inflammatory cell infiltration by flow cytometry, immunohistochemistry, semiquantitative real-time reverse transcriptase polymerase chain reaction, and detection of fluorescence-labeled IH11128 in kidney tissues.
