# Supplementary material for: The complement factor 5a receptor 1 has a pathogenic role in chronic inflammation and renal fibrosis in a murine model of chronic pyelonephritis
Source: Kidney Int. 2016 Sep;90(3):540–54. doi: 10.1016/j.kint.2016.04.023 (PMC4996631; doi:10.1016/j.kint.2016.04.023)
Supplement: Supplementary Methods — The following additional information for methods is given in supplementary methods: materials, cell cultures, assessment of effects of C5a/C5aR1 interaction on cytokine production by RTECs and MO/MΦs in response to bacteria stimulation, assessment of bacterial uptake and intracellular killing by MO/MΦs, assessment of renal inflammatory cell infiltration by flow cytometry, immunohistochemistry, semiquantitative real-time reverse transcriptase polymerase chain reaction, and detection of fluorescence-labeled IH11128 in kidney tissues. [file mmc1.doc]

**Supplementary methods**

**Materials**

We used the following reagents and materials: cysteine lactose electrolyte deficient (CLED) agar plates (Oxoid Ltd, Basingstoke, UK); cell culture medium, fetal calf serum (FCS), insulin-transferrin-selenium solution, gentamicin, penicillin, streptomycin, Triton X-100, EDTA, gelatin (Invitrogen, Paisley, UK); hydrocortisone, tri-iodothyronine, Tetramethylrhodamine isothiocyanate mixed isomers (TRITC) (Sigma-Aldrich, Dorset, UK); type II collagenase (Worthington Bio. Co. Lakewood, USA); Oligo(dT), deoxynucleotide triphosphates and moloney murine leukemia virus reverse transcriptase (Promega, Southampton, UK). DyNAmo HS SYBR Green, Coomassie (Bradford) protein assay kit (Thermo Fisher, Loughborough, UK); **fluorescein labelled Lotus Tetragonolobus Lectin (LTL)** (Vector Laboratories, Peterborough, UK); purified mouse C5a (Hycult Biotech, Uden, the Netherlands); rat anti-mouse CD45 (30-F11, APC or FITC), Ly6G (1A8, PE), Ly6C (HK1.4, PE/cy7), CD11b (M1/70, FITC or PE/cy7), Gr-1 (RB6-8C5, APC), purified rat anti-mouse CD45 (30-F11) and F4/80 (BM8) (BioLegend, San Diego, USA); HRP-conjugated rabbit anti-rat was from DAKO (Ely, UK); 4',6-diamidino-2-phenylindole, dihydrochloride (DAPI) (Life Sciences, Paisley, UK).

**Cell cultures**

Primary RTEC cultures were prepared from kidneys of normal C57BL/6 mice as described previously [2]. After 7-10 d, confluent layers of RTEC were used for assays for cytokine production. MO/MΦ were prepared from the peritoneal lavage of C57BL/6 mice 3 days after i.p. injection of 1 ml of 3% thioglycollate. The adherent cells were used for assays for cytokine production and phagocytic function.

**Assessment of effects of C5a/C5aR1 interaction on cytokine production by RTEC and MO/MΦ in response to bacteria stimulation**

Confluent layers of RTEC grown on 24-well plates or 2 × 10^6^/well MO/MΦ seeded in 24-well plates were incubated with C5a (10nM) and heat killed IH11128 (2 × 10^6^/well), either alone or combined, in a total volume of 1 mL at 37^o^C for 24 h, followed by RT-PCR.

**Assessment of bacterial uptake and intracellular killing by MO/MΦ**

MO/MΦ were incubated with or without C5a (10 and 50nM) for 4h and then co-cultured with IH11128 [MOI = 5:1; 1×10^6^ bacteria and 2×10^5^ cells] for 45 minutes at 37°C. To assess bacterial uptake by MO/MΦ, after washing, the MO/MΦ were incubated with gentamicin (100 μg/mL) for 1 h to kill extracellular bacteria then lysed with sterile H_2_O. To assess intracellular killing of phagocytosed E. coli, following gentamicin treatment, the MO/MΦ were further cultured for an additional 24 h in the absence of gentamicin and then lysed with sterile H_2_O. The lysates generated from above two procedures were plated out on CLED plates, colonies were counted and results were expressed as CFU per well.

Assessment of renal inflammatory cell infiltration by flow cytometry

Single renal cell suspension was prepared using the method described previously, with modifications [1]. Kidneys were weighed, minced and incubated with collagenase D (0.75 mg/mL) for 10 min at 37^o^C with gentle agitation. The collagenase was inactivated with an equal volume of DMEM-F12 containing 10% FCS. The digested tissue mixture was then passed through a 40 µm nylon sieve to remove tissue debris. The cell segments were collected and treated with red cell lysis buffer to remove remaining RBC. The cell pellet was washed and re-suspended in PBS containing 1% BSA, followed by flow cytometric analysis. The cells were pre-incubated with FcR blocking antibody (CD16/32) and then stained with fluorochrome-conjugated monoclonal antibodies or the appropriate isotype control antibodies at 4°C for 20 min. In order to quantify absolute cell counts in kidney tissue, we used CountBright™ absolute counting beads in our flow cytometry assays according to the instructions of the manufacturer. All flow cytometric analysis was performed using Calibur Flow Cytometer (BD Biosciences) and Flowjo software (Tree Star, OR, USA).

**Immunohistochemistry**

Frozen sections (4 μm) were air-dried and then acetone-fixed. Indirect immunohistochemical staining for leukocytes and MO/MΦ was performed using rat anti-mouse CD45 and - F4/80 antibody and HRP-conjugated rabbit anti-rat polyclonal antibody. Stained kidney sections were visualized under light microscopy (Nikon 50i) and photographed at ×200 and ×400 magnifications. CD45+ or F4/80+ cells were quantified by counting the number of positively stained cells. 10 cortical-medullar junction viewing fields randomly selected for each murine kidney were examined and expressed as a number of cells per filed (0.04mm^2^). The quantitative analysis was performed in a blinded fashion by two experienced persons.

**Semi-quantitative real time RT-PCR**

Total RNA extraction from tissues and cells, reverse transcription reaction, and the PCR were performed as previously described [4]. The relative gene expression was analyzed using the 2^-ΔΔ^*^C^*^T^ method [3] and expressed as 2^-ΔΔ (Ct)^, where Ct is cycle threshold, ΔΔ (Ct) = testing samples Δ (Ct) - control samples Δ (Ct); Δ (Ct) = testing gene (Ct) -18s (Ct). The control samples are normal kidney tissues. The testing samples are infected kidney tissues. The information for primer sequences is given in supplementary table S1.

**Detection of fluorescently labeled IH11128 in kidney tissues**

For labeling bacteria, overnight cultures of IH11128 were washed and re-suspended in PBS (10^9^ cfu/mL). Tetramethylrhodamine (TRITC) was added to a final concentration of 1mg/ml and incubated for 3h with gentle shaking in the dark. Bacteria were vigorously washed to remove unbound TRITC. For detection of bacterial colonization in kidney sections, the labelled IH11128 (5 × 10^8^ cfu in 50μl PBS) were injected into the bladder *per* urethra. Mice were killed at day 2 post-infection, frozen sections of infected kidneys were stained with DAPI and **fluorescein labelled** LTL (a marker for proximal tubular cells), and viewed and imaged with the confocal laser scanning microscope system (Leica TCS SP8). Bacterial colonies were counted and results were expressed as number of colonies per field. 15 viewing fields, randomly selected from inner medullar, cortical-medullar junction and out cortex areas (5 fields for each area) at ×200 magnification for each kidney, were examined.

**Supplementary Table S1**

# PCR primer sequences and product sizes

| Primer* | **Oligonucleotide Sequence**  **(5’ → 3’)** | **Product Size (bp)** | **Gene bank code** |
| --- | --- | --- | --- |
| 18S-1 | ATC CCT GAG AAG TTC CAG CA | 153 | NM_011296.1 |
| 18S-2 | CCT CTT GGT GAG GTC GAT GT |  |
| TNF-α-1  TNF-α-2 | TGA GCA CAG AAA GCA TGA TCC  GCC ATT TGG GAA CTT CTC ATC | 200 | NM_013693.3 |
| IL6-1  IL6-2 | gtt ctc tgg gaa atc gtg ga  GGA AAT TGG GGT AGG AAG GA | 339 | NM_031168 |
| KC-1 | TGA AGC TCC CTT GGT TCA GA | 361 | NM_008176.3 |
| KC-2 | TGC ACT TCT TTT CGC ACA AC |  |
| MCP-1-1  MCP-1-2 | GGC TCA GCC AGA TGC AGT TA  ATT TGG TTC CGA TCC AGG TT | 219 | NM_011333.3 |
| CCR2-1  CCR2-2 | CCT GCA AAG ACC AGA AGA GG  TCC AAG CTC CAA TTT GCT TC | 254 | NM_009915.2 |
| TGF-β-1 | AAT ACG TCA GAC ATT CGG GAA | 640 | NM_011577 |
| TGF-β-2 | CCG GGT TGT GTT GGT TGT AGA G |  |  |
| PDGF-1  PDGF-2 | TAT GAA ATG CTG AGC GAC CA  GAT CGA TGA GGT TCC GAG AG | 250 | ENSMUST00000000500 |
| HGF-1  HGF-2 | TCC CTG AAA AGA CCA CTT GC  ATC TCC CTC ACA TGG TCC TG | 200 | NM_010427.5 |
| Collagen I -1  Collagen I -2 | TGA CTG GAA GAG CGG AGA GT  GTT CGG GCT GAT GTA CCA GT | 151 | OTTMUST00000004184 |
| Collagen IV -1  Collagen IV -2 | CTG GGA AAG ACG GTG AAA AA  TGG TGC CTA TCA CAG TTC CA | 147 | ENSMUST00000033898 |
| Fibronectin-1  Fibronectin-2 | GAA GTC GCA AGG AAA CAA GC  GCA TCG TAG TTC TGG GTG GT | 393 | NM_010233 |
| α-SMA-1  α-SMA-2 | GCT GGT GAT GAT GCT CCC A  GCC CAT TCC AAC CAT TAC TCC | 81 | NM_007392.3 |
| Vimentin-1  Vimentin-2 | CGG CTG CGA GAG AAA TTG C  CCA CTT TCC GTT CAA GGT CAA G | 124 | NM_011701.4 |

* Primer-1 is identical to the coding strand; primer-2 is complementary to the coding strand.

**Reference List**

**1. Dong X, Swaminathan S, Bachman LA *et al.*: Resident dendritic cells are the predominant TNF-secreting cell in early renal ischemia-reperfusion injury. *Kidney Int* 71:619-628, 2007**

**2. Li K, Patel H, Farrar CA *et al.*: Complement activation regulates the capacity of proximal tubular epithelial cell to stimulate alloreactive T cell response. *J Am Soc Nephrol* 15:2414-2422, 2004**

**3. Livak KJ, Schmittgen TD: Analysis of relative gene expression data using real-time quantitative PCR and the 2(-Delta Delta C(T)) Method. *Methods* 25:402-408, 2001**

**4. Peng Q, Li K, Smyth LA *et al.*: C3a and C5a promote renal ischemia-reperfusion injury. *J Am Soc Nephrol* 23:1474-1485, 2012**
